# Inhibition of microbial sulfate reduction in a flow-through column system by (per)chlorate treatment

**DOI:** 10.3389/fmicb.2014.00315

**Published:** 2014-06-26

**Authors:** Anna Engelbrektson, Christopher G. Hubbard, Lauren M. Tom, Aaron Boussina, Yong T. Jin, Hayden Wong, Yvette M. Piceno, Hans K. Carlson, Mark E. Conrad, Gary Anderson, John D. Coates

**Affiliations:** ^1^Department of Plant and Microbial Biology, University of California, BerkeleyBerkeley, CA, USA; ^2^Lawrence Berkeley National Laboratory, Earth Sciences DivisionBerkeley, CA, USA

**Keywords:** perchlorate reduction, petroleum microbiology, souring, sulfate reduction, sulfur

## Abstract

Microbial sulfate reduction is a primary cause of oil reservoir souring. Here we show that amendment with chlorate or perchlorate [collectively (per)chlorate] potentially resolves this issue. Triplicate packed columns inoculated with marine sediment were flushed with coastal water amended with yeast extract and one of nitrate, chlorate, or perchlorate. Results showed that although sulfide production was dramatically reduced by all treatments, effluent sulfide was observed in the nitrate (10 mM) treatment after an initial inhibition period. In contrast, no effluent sulfide was observed with (per)chlorate (10 mM). Microbial community analyses indicated temporal community shifts and phylogenetic clustering by treatment. Nitrate addition stimulated *Xanthomonadaceae* and *Rhizobiaceae* growth, supporting their role in nitrate metabolism. (Per)chlorate showed distinct effects on microbial community structure compared with nitrate and resulted in a general suppression of the community relative to the untreated control combined with a significant decrease in sulfate reducing species abundance indicating specific toxicity. Furthermore, chlorate stimulated *Pseudomonadaceae* and *Pseudoalteromonadaceae*, members of which are known chlorate respirers, suggesting that chlorate may also control sulfidogenesis by biocompetitive exclusion of sulfate-reduction. Perchlorate addition stimulated *Desulfobulbaceae* and *Desulfomonadaceae*, which contain sulfide oxidizing and elemental sulfur-reducing species respectively, suggesting that effluent sulfide concentrations may be controlled through sulfur redox cycling in addition to toxicity and biocompetitive exclusion. Sulfur isotope analyses further support sulfur cycling in the columns, even when sulfide is not detected. This study indicates that (per)chlorate show great promise as inhibitors of sulfidogenesis in natural communities and provides insight into which organisms and respiratory processes are involved.

## Introduction

Although non-traditional energy sources such as bioethanol, solar, and wind will increase over the coming decades, it is predicted that these will account for less than 10% of total demand by 2030 (US Department of Energy: www.eia.doe.gov/oiaf/ieo/index.html). As such, global reliance on fossil energy and oil recovery will likely continue to dominate in the near future. An important aspect of oil recovery is control of reservoir bio-souring, which is the result of *in situ* hydrogen sulfide (H_2_S) biogeneration, typically after initiation of secondary recovery processes involving injection of sulfate-rich seawater (Youssef et al., 2009; Gieg et al., 2011).

As the primary cause of industrial gas inhalation deaths in the US (https://www.osha.gov/SLTC/hydrogensulfide/hazards.html), the generation of H_2_S by sulfate reducing microorganisms (SRM) poses significant health (Fuller and Suruda, 2000) and environmental risks and results in a variety of oil recovery problems, including contamination of crude oil, metal corrosion, and precipitation of metal sulfides that plug pumping wells (Vance and Thrasher, 2005). Representatives within the domains Archaea and Bacteria have been identified as SRM contributing to souring in oil reservoirs. As such, targeting of specific species, genera, or even phyla for inhibition is of limited value. Because of this, efforts have focused on mechanisms by which the dissimilatory sulfate-reducing metabolism can be inhibited.

Intensive research has centered on thermodynamic inhibition of SRM by the addition of nitrate to the injection waters (Voordouw et al., 2009; Youssef et al., 2009; Hubert, 2010; Gieg et al., 2011). Thermodynamic considerations indicate that microbial nitrate reduction is energetically more favorable than sulfate reduction and should therefore occur first (Lovley and Chapelle, 1995). For example the Gibbs free energy for the anaerobic degradation of toluene coupled to nitrate reduction (Δ G^o'^ = −3529 kJmol^−1^ toluene) is significantly higher than that coupled to sulfate reduction (Δ G^o'^ = −179 kJmol^−1^ toluene) (Rabus and Heider, 1998). While bio-competitive exclusion may operate in some systems, the favorable thermodynamics of nitrate reduction does not exclude the prospect that sulfate reduction can still occur if the electron donor is saturating (Lovley and Goodwin, 1988), as is the case in an oilfield. The electron acceptor being consumed at any specific location is controlled by the respective concentrations of the electron donor and individual electron acceptors (Lovley et al., 1995; Coates et al., 1996b, 2001; Christensen et al., 2000). Thus, as nitrate depletes in the near-well environment, or in microenvironments within the reservoir matrix, sulfate reduction can still be active deeper in the reservoir (Voordouw et al., 2009; Callbeck et al., 2011). While nitrite, a transient intermediate of nitrate reduction, can have a significant inhibitory effect on SRM (Callbeck et al., 2013), it is also chemically and biologically labile and has a limited half-life in a reduced reservoir matrix. Furthermore, the Nrf nitrite reductase is widely distributed amongst the known SRM, and has been demonstrated to provide an intrinsic defense mechanism against nitrite toxicity (Greene et al., 2003). Finally, nitrate addition also enriches for lithoautotrophic sulfur oxidizing nitrate reducing bacteria that oxidize sulfide to sulfate and mask the activity of active SRM (Gevertz et al., 2000). As such, in order to ensure inhibition of active sulfate reduction it is imperative to maintain a nitrate concentration in injection fluids high enough to prevent nitrate depletion during its residence in the formation and biogenesis of large quantities of nitrite (Callbeck et al., 2013). Under these conditions, nitrate addition can successfully impede SRM activity (Sunde and Torsvik, 2005) although not necessarily completely attenuate it (Callbeck et al., 2013). However, this requires the addition of saturating amounts of nitrate, which is not always financially feasible or logistically possible.

Here we investigate a novel strategy to biologically control biogenic H_2_S generation based on the introduction of (per)chlorate into injection waters and the stimulation of the activity of dissimilatory (per)chlorate reducing bacteria (DPRB) in oil reservoirs. The advantage of this approach is that in addition to thermodynamic preference (*E*^o'^ = +797 mV and +792 mV for the biological couple of ClO_4_^−^/Cl^−^ and ClO_3_^−^/Cl^−^, respectively) relative to sulfate reduction (*E*^o'^ = −217 mV), previous studies (Postgate, 1952; Baeuerle and Huttner, 1986) demonstrated that high concentrations of (per)chlorate may be directly and specifically inhibitory to microbial sulfate-reduction. This is in contrast to nitrate inhibition which is primarily due to the production of the toxic transient intermediate nitrite (He et al., 2010). An additional aspect of souring treatment by (per)chlorate is based on the fact that while these compounds are kinetically stable in the presence of sulfide (Gregoire et al., 2014), all DPRB tested to date innately oxidize H_2_S rapidly (Bruce et al., 1999; Coates et al., 1999; Coates and Achenbach, 2004), preferentially utilizing it over labile organic electron donors and producing benign elemental sulfur as the sole end product of the metabolism (Gregoire et al., 2014).

In our studies, advective flow column systems were packed with marine sediment through which we pumped seawater to assess the comparative effectiveness of nitrate, perchlorate, and chlorate in controlling souring. The progress of souring and the utilization of the added treatments (nitrate, chlorate, or perchlorate) was monitored by analyzing influent and effluent geochemistry and sulfur isotopes, while community 16S ribosomal RNA gene analysis was used to gain insight into the shifts in microbial community composition.

## Materials and methods

### Column setup and operation

Triplicate flow-through columns were constructed from sealed 50 mL glass syringes packed with a mixture of about 50% San Francisco Bay sediment (microbial inoculum) and about 50% glass beads (70–100 μm diameter, used to improve column permeability). The constructed columns were flooded with autoclaved anoxic (boiled and degassed with N_2_) San Francisco Bay water (19–33 mM sulfate concentration) containing 1 g.L^−1^ yeast extract as a non-selective labile carbon source. Treatments consisted of 10 mM sodium nitrate, 10 mM sodium chlorate, 10 mM sodium perchlorate, or a no treatment control. The treatment concentration was briefly reduced to 5 mM for all three treatments at day 35 for a period of 3 days and then returned to 10 mM to study the impact of lower treatment concentration on the column geochemistry. The control columns were unchanged during this time period. All four treatments were run with triplicate columns and identical flow rates. During the initial 28 days of the study, the columns were continuously flooded at 0.1 mL.min^−1^ for 2 days (estimated retention time 2.78 h) with subsequent 2 days of no feed. After 20 days the flow rate was decreased to 0.025 mL.min^−1^ (2 days of flow at 0.025 mL/min followed by 2 days of no flow). Continuous flow at 0.025 mL.min^−1^ was established from day 28 with an estimated retention time of 11.11 h and a variance of less than 1% (±0.00025 mL.min^−1^). The columns were run for a total of 51 days. As total flow was very similar between all columns regardless of treatment, a cross-comparison of column treatments could be reasonably achieved.

### Analytical techniques

Nitrate, chlorate, and sulfate anions were quantified by ion chromatography on a Dionex IC 1500 using an AS9-HC anion-exchange column with a 9 mM sodium carbonate mobile phase at a flow rate of 1 mL.min^−1^. Perchlorate was quantified on a Dionex IC 2100 equipped with an AS16-HC anion-exchange column (Dionex IC2100) with a 25–65 mM potassium hydroxide gradient at a flow rate of 1 mL.min^−1^. Sulfide concentrations were quantified using a Cline assay (Cline, 1969) read at 660 nm on a Varian Cary 50 Bio spectrophotomer equipped with a Cary 50 MPR microplate reader.

Sulfur isotope analysis of dissolved sulfate was conducted on samples selected from one replicate column of each treatment. Sulfate was first precipitated as barium sulfate by adding excess barium chloride. The precipitate was rinsed three times in deionized water before being dried for analysis and sulfur isotope ratios were measured using a Eurovector 3028 elemental analyzer in helium continuous flow mode with a GV Isoprime isotope ratio mass spectrometer. Instrumental precision as assessed on external standards was ±0.2‰. Sulfur isotope ratios are reported in standard delta notation, δ^34^S = (R_sample_/R_std_ −1) × 1000, where *R* = ^34^S/^32^S, and the value is reported in per mil (‰) units relative to the Canyon Diablo Troilite standard (R_std_ = 0.0441216).

### Phylochip

To characterize changes in the microbial community due to the various treatments sediment samples were collected from the top (outlet) of the columns, DNA was isolated from the initial columns before flow began (designated inoculum) and from each of the triplicate columns for each treatment at four other time points (Days 31, 38, 42, and 51) using a Mo Bio PowerSoil DNA isolation kit (Mo Bio Laboratories, Inc., Carlsbad, CA) following the manufacturers protocol. DNA was quality assessed by agarose gel electrophoresis. PCR amplification was conducted as previously described (Wrighton et al., 2008); the amplifications used 1 ng of gDNA as template and were performed over an 4-gradient annealing temperature (4 PCR reactions were performed for each sample within a 50–56 C gradient and pooled) using non-degenerate primers 27f (5′-AGAGTTTGATCCTGGCTCAG-3′) and 1492r (5′-GGTTACCTTGTTACGACTT). PCR amplifications were restricted to 25 cycles. PCR reactions were prepared for PhyloChip analysis and data were treated as previously described (Handley et al., 2012).

### Community analyses

PhyloChip data was analyzed using PRIMER 6 (PRIMER-E Ltd, Plymouth, UK) and Excel (Microsoft Co, Redmond, WA). OTU data was square root transformed and normalized. Hierarchical clustering based on group average (the mean distance apart of two groups, averaging over all between group pairs) and nonmetric multidimensional scaling (nMDS), both based on Bray-Curtis similarity matrices were used to assess clustering amongst the samples. Both had low stress values (calculated on a scale of 0–1) indicating that the plots were indeed a good representation of the data. Similarity Profile (SimProf) was used as a statistical measure to determine significance to the groupings identified in the Hierarchical clustering at a 5% significance level.

Similarity percentage (SIMPER) based on the Bray-Curtis similarity matrix was used to determine the OTUs contributing to the top 10% of differences between the groups. These OTUs were sorted by family and phylum. Relative abundance values for all OTUs within a family or phylum were summed. The total abundance differences between groups for each family were then calculated for each treatment.

### Community richness and diversity

Relative richness (measure of the number of different OTUs in a community) was calculated using OTU presence/absence calls from the PhyloChip data. Relative diversity and evenness were calculated using Shannon's diversity and equitability measures, respectively. Evenness is a measure of equality of the community (the relative abundance of the different OTUs present in the community). These calculations were based on the intensity values from the PhyloChip. Because PhyloChip abundance is based on hybridization scores (intensity) relative, not absolute, diversity, and evenness were calculated.

## Results

### Column influent and effluent treatment ion dynamics

**Figure 1:**
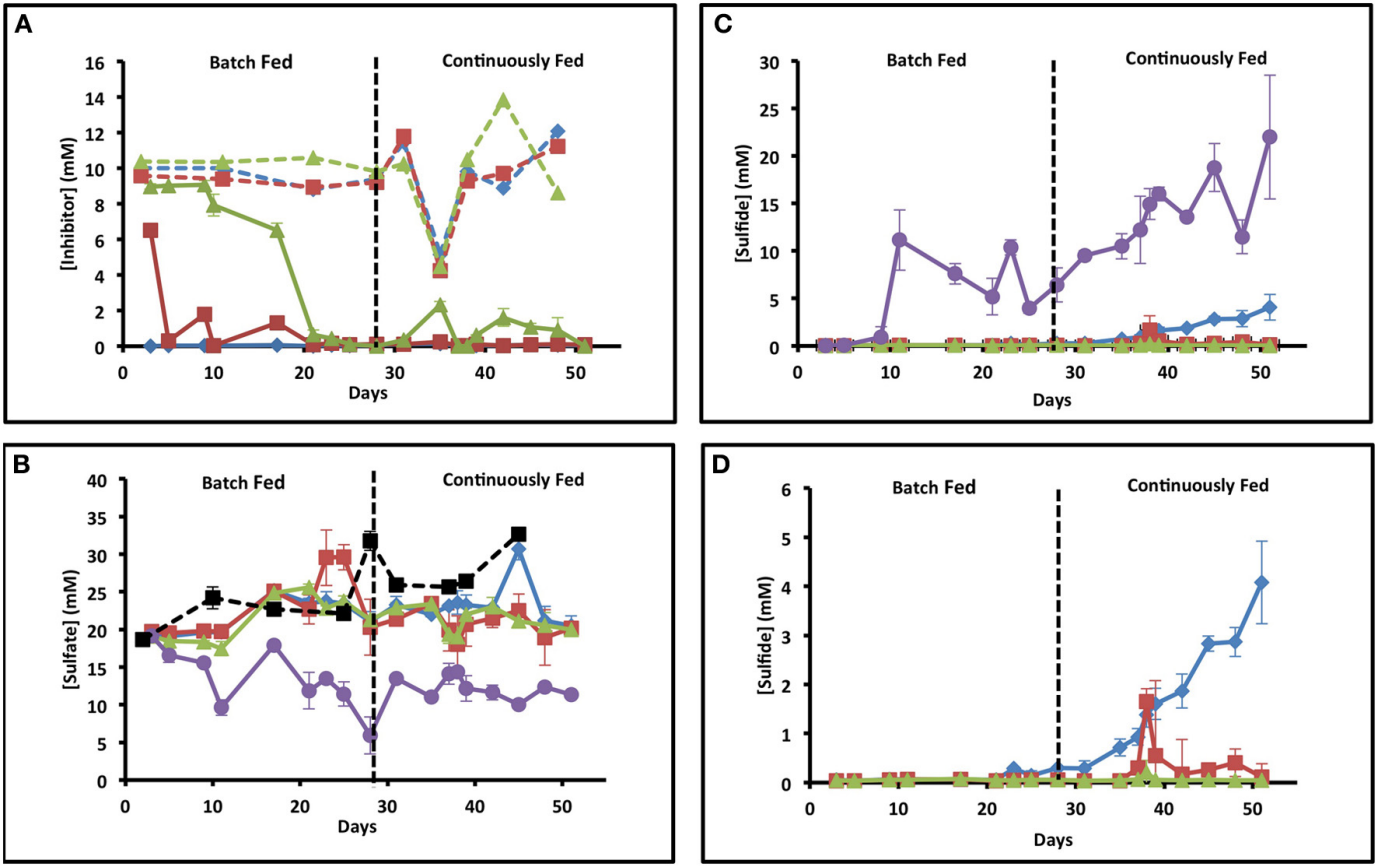
**Geochemistry of the influent and effluent of each set of columns**. X axis is time in days and y axis is concentration. Error bars represent standard deviation of samples from triplicate columns. Blue diamonds represent samples from nitrate columns, red squares represent samples from chlorate columns, green triangles represent samples from perchlorate columns, and purple circles represent samples from the no treatment columns. **(A)** Influent (dashed lines) and effluent (solid lines) concentrations of the three treatments over time. **(B)** Influent (dashed black line with black squares) and effluent (solid lines) sulfate concentrations over time in days. **(C)** Effluent concentrations of sulfide over time in all the columns. **(D)** Blowup of a portion of **(C)** focusing on just the treated columns.

In order to examine the effect of (per)chlorate on souring in comparison to nitrate in a dynamic sediment system, we monitored the influent and effluent ion concentrations of triplicate up-flow columns for each treatment. Influent nitrate, chlorate, and perchlorate concentrations were kept constant (~10 mM) throughout the study except for a brief period (day 35–38) when the treatment concentration was decreased by 50% to 5 mM (Figure 1A). Of note, nitrate was never detectable in the effluent from the nitrate treated columns suggesting a rapid adaptation of the microbial community and complete nitrate depletion within the column matrix (Figure 1A). Similarly, chlorate concentrations rapidly dropped in the effluent of the chlorate columns and hovered around 1 mM during the fed batch phase of the study but rapidly decreased to below detection once continuous feeding was established (days 28–51) (Figure 1A). Although (per)chlorate is relatively stable in the presence of sulfide (Gregoire et al., 2014), chlorate is chemically reactive with Fe(II) according to ClO_3_^−^ + 6Fe^2+^ + 9H_2_O → 6FeOOH + Cl^−^ + 12H^+^ (Figure S1). The ferric oxyhydroxide formed (FeOOH) can subsequently be reduced by sulfide according to 6FeOOH + 3HS^−^ + 15H^+^ → 6Fe(II) + 3S^o^ + 12H_2_O resulting in redox cycling of the iron as a catalyst and abiotic sulfide oxidation coupled to chlorate reduction. The rapid decrease in effluent chlorate concentrations is probably a combination of both abiotic consumption catalyzed by the Fe(II) content of the column matrices and microbial community adaptation resulting in microbial chlorate respiration. In contrast to both nitrate and chlorate, perchlorate concentrations remained above 6 mM for the first 17 days of the study and then decreased to below detection by day 21 (Figure 1A). The difference in the rate of chlorate and perchlorate removal is consistent with the chemical stability of perchlorate relative to chlorate even in the presence of Fe(II) (Figure S1) (Urbansky, 1998, 2002; Urbansky and Brown, 2003) preventing its abiotic removal. As such, its depletion would primarily be driven by microbial adaptation and respiration. As perchlorate is not an abundant electron acceptor in the majority of environments, including the marine sediments from which we obtained our microbial inoculum, (Rajagopalan et al., 2009) slow adaptation of the resident microbial community to perchlorate respiration would be expected.

### Column influent and effluent sulfate and sulfide dynamics

Throughout operation, some variation in influent sulfate concentrations (19–33 mM) occurred due to tidal mixing with freshwater sources (Sacramento River) at the location of water collection in San Francisco Bay (Figure 1B). In the absence of any column treatment, effluent sulfate concentrations steadily dropped throughout the first 10 days of operation and stabilized at a concentration of 9.64 ± 1.04 mM (mean ± 1σ, *n* = 3) after 11 days, which is equivalent to approximately 40% removal (Figure 1B, purple line). In support of this sulfide concentrations showed a steady increase from day 10 (Figure 1C, purple line) to a maximum of 21.99 ± 6.52 mM (mean ± 1σ, *n* = 3) by the final day of the study (day 51). During the last 10 days of column operation the average effluent sulfide concentration was 16.44 ± 4.83 mM, which stoichiometrically balanced the sulfate removal (18.2 ± 4.55 mM) during the same timeframe.

In contrast to the no treatment controls, sulfate concentrations in the effluent of all treated columns showed no significant variation from the influent on a daily basis over the initial 23 days of operation indicating no apparent net sulfate removal (Figure 1B; *t*-test, *p*-value = 0.41). In support of this, sulfide concentrations in the effluent remained below detection (<0.1 mM) during this timeframe (Figure 1C). Unexpectedly, although influent nitrate concentrations (10 mM) remained constant, sulfide breakthrough was observed at day 23 in all nitrate columns and measurable sulfide increased steadily to 4.08 ± 1.36 mM by day 51 (Figure 1D). In contrast, no sulfide breakthrough was observed in the (per)chlorate treated columns except for a short period when the influent treatment concentrations were decreased to 5 mM (from 10 mM) (day 35–38) (Figure 1D). In this instance sulfide production was transient and peaked at 1.65 ± 1.53 mM on day 38 for the chlorate columns, and 0.21 ± 0.24 mM on day 38 for the perchlorate columns (sulfide breakthrough was evident in only one of the three perchlorate columns), decreasing back to below detection by day 39 in both instances.

### Sulfur isotope analysis

**Figure 2:**
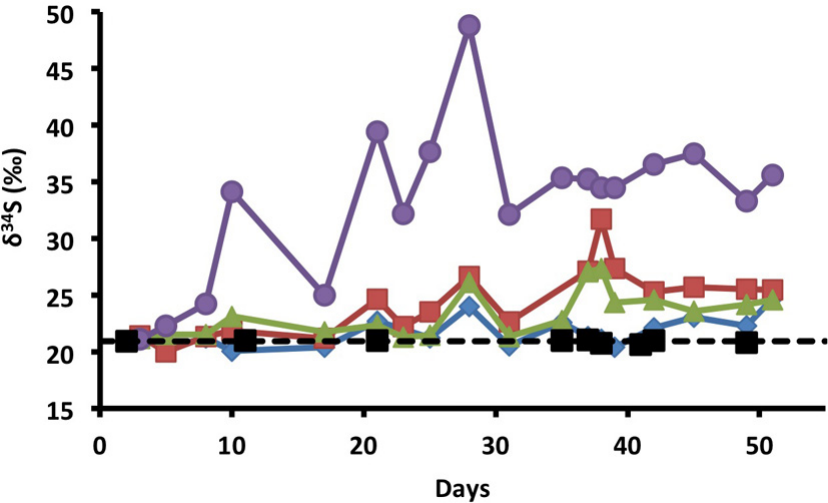
**Variation in sulfur isotope ratios of dissolved sulfate with time for influent (black squares and dashed line); and for effluent, nitrate (blue diamonds), chlorate (red squares), perchlorate (green triangles), and no treatment control samples (purple circles)**.

Sulfur isotope ratios of dissolved sulfate were used to gain further insight into sulfur cycling within the columns. Microbial sulfate reduction results in a well-established shift in the δ^34^S values of residual sulfate, leading to higher δ^34^S values as the fraction of initial sulfate decreases (Kaplan and Rittenberg, 1964; Brüchert, 2004; Brunner and Bernasconi, 2005). In contrast, the sulfide produced has lower δ^34^S values than the initial sulfate. The variation in sulfate δ^34^S with time was examined for one replicate column of each treatment (Figure 2). It is noteworthy that the influent sulfate had a stable δ^34^S value (20.9 ± 0.1‰, mean ± 1σ, *n* = 9), despite variations in the influent sulfate concentration (Figure 1B) providing a reliable baseline against which effluent δ^34^S values were evaluated. In the control columns, effluent sulfate δ^34^S values rapidly increased to a maximum of 48.8‰ on day 28 associated with SRM activity. Effluent sulfate δ^34^S values were highly variable before day 28 (31.6 ± 9.3‰, *n* = 9), with high values corresponding to samples taken after periods of no-flow alongside a greater extent of sulfate reduction within the columns (Figure 1) and lower values corresponding to flow periods (Figure 2). After day 28, stable δ^34^S values (35.0 ± 1.6‰, *n* = 9) reflect the period of stable continuous flow and a general community δ^34^S fractionation value.

In contrast to the control columns, the lower extent of sulfate reduction in the treated columns resulted in lower δ^34^S values (Figure 2). Of note, slight increases in effluent δ^34^S above influent values were seen in all treatment columns before measurable sulfide was apparent (Figures 1C, 2) indicating that some sulfate reduction occurred even in the presence of the treatment. Visual observations of a blackening of the column matrix, especially in the nitrate columns, suggest that this temporal offset was likely due to reaction of sulfide with iron in the bay sediment, thereby scavenging H_2_S from solution as iron sulfide precipitates (Morse et al., 1987). A comparison of the nitrate and (per)chlorate columns indicated that although effluent sulfide concentrations were present in the nitrate treatment and absent in the (per)chlorate treatment, effluent sulfate δ^34^S values were comparable for each of the individual treatments (chlorate δ^34^S = 24.3 ± 3.0‰; mean ± 1σ, *n* = 17; perchlorate 23.3 ± 2.0‰; *n* = 18; nitrate 21.7 ± 1.3‰; *n* = 18). This discrepancy between the (per)chlorate and nitrate treatment is consistent with active redox cycling between sulfide and sulfate in the nitrate columns in which the isotopic sulfate-S signature is masked by the complete microbial oxidation of sulfide to sulfate by sulfur-oxidizing nitrate-reducing bacteria as previously observed (Telang et al., 1997).

### Community analyses

PhyloChip (Desantis et al., 2007; Wrighton et al., 2008; Mendes et al., 2011; Handley et al., 2012) microbial community analysis was performed on four replicates of the initial inoculum material and on each triplicate column on four separate temporal samples throughout the study. Sampling dates were chosen that covered the period during which the columns were under continuous flow conditions.

**Figure 3:**
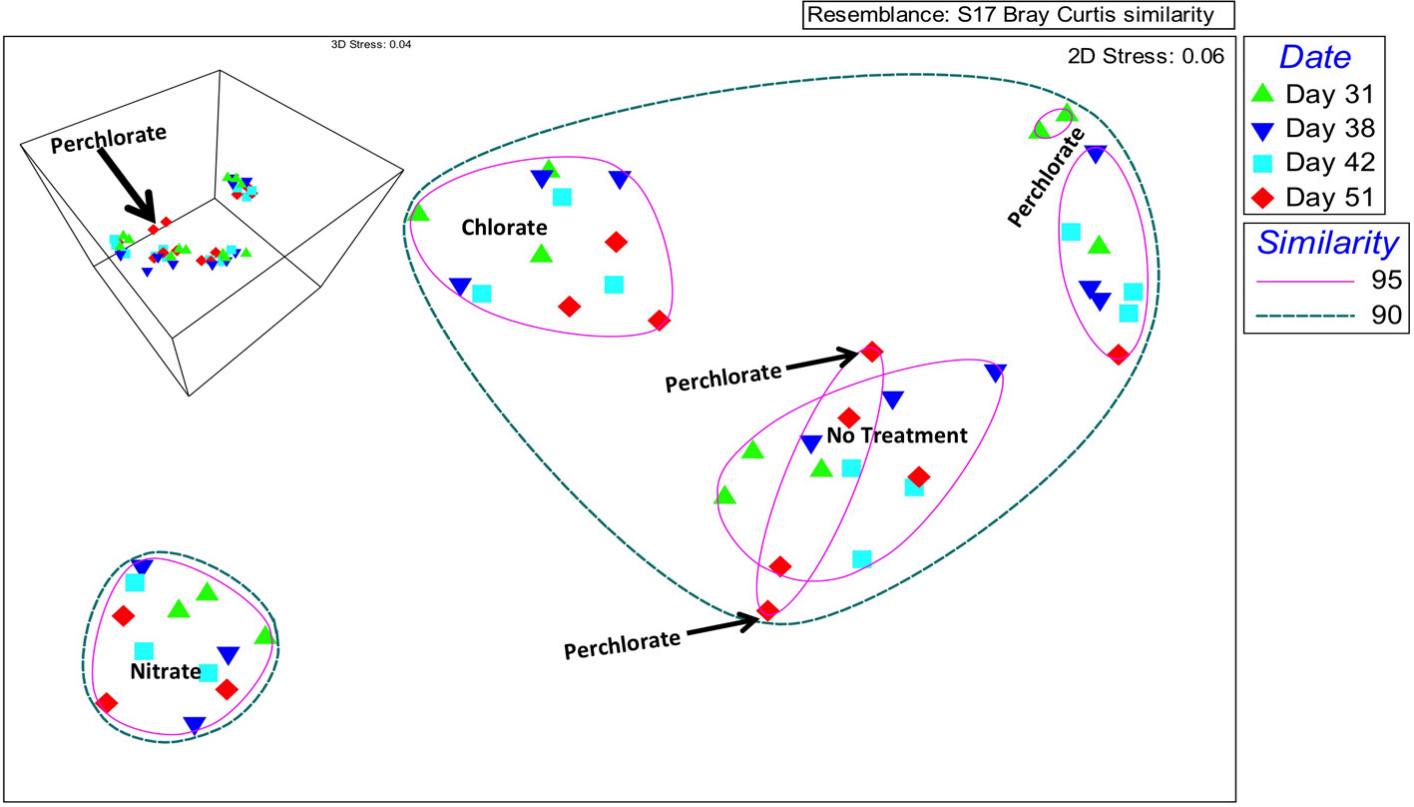
**Nonparametric multidimensional scaling of community data based on a Bray-Curtis similarity matrix**. Circles represent percent similarity based on hierarchical clustering and colored symbols represent sampling day. The same plot in three dimensions is inset in the upper left hand corner.

Community changes at the OTU level were investigated by nonparametric Multidimensional Scaling (nMDS) indicated an overall grouping by treatment (Figure 3). In two-dimensional space, the chlorate, nitrate, and no treatment temporal samples all clustered within individual treatment groups at 95% similarity. In contrast, at this similarity percentile the perchlorate samples grouped into three independent temporal phases (day 31; days 38, and 42; day 51), consistent with an extended adaptation period to perchlorate relative to nitrate or chlorate (Figure 3), an observation supported by the geochemical data (Figure 1A). In the two dimensional representation of the nMDS, two perchlorate samples from day 51 appear to group with some of the no treatment samples at 95% similarity but in three dimensional space they form an independent group (Figure 3 insert, black arrow). At a 90% similarity level the perchlorate, chlorate, and no treatment samples all clustered together with the nitrate clustering independently indicating that nitrate had a more drastic effect on the community composition than the perchlorate or chlorate. This is probably due to the rapid outgrowth of a nitrate respiring community relative to a more slowly adapting (per)chlorate respiring community.

Relative diversity and evenness calculations indicated that the perchlorate treatment had the highest relative evenness (Figure S2). The perchlorate samples also had the lowest relative richness based upon presence-absence data. These results suggest that perchlorate had a general uniform effect on the microbial community as a whole and did not stimulate any specific population to a large extent although it may have selective inhibited some members. In contrast, nitrate treatment resulted in the highest relative diversity and the lowest relative evenness (Figure S2). Interestingly, the relative richness was similar to that in the initial and no treatment samples suggesting that nitrate did not have a broad inhibitory effect. Rather, the low evenness suggests that nitrate was in fact a stimulant of some microbial populations pre-existing within the initial community.

**Figure 4:**
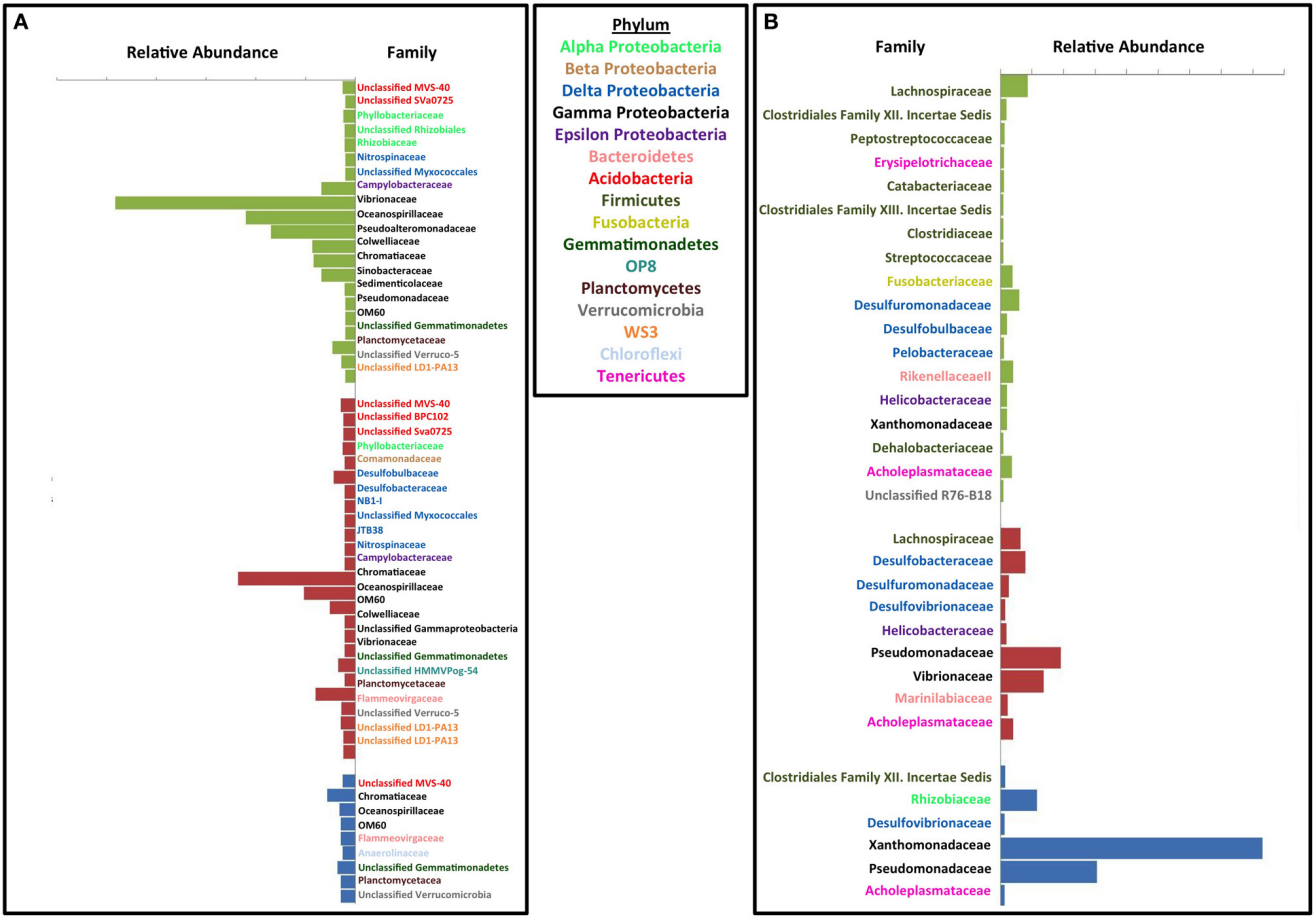
**Effect of each treatment on microbial community compared to the initial innoculum**. Similarity percentage (SIMPER) was used to determine the OTUs that contribute to the top 10% of difference between each set of treatment samples and the initial inoculum samples. Average abundance for the OTUs belonging to the same family were added together. Abundance on the x axis is the difference between average abundance for samples from the corresponding treatment and the initial inoculum samples. Each plot is labeled by family on the y axis and the family names are color coded by phylum. Green bars represent perchlorate treated columns, red bars represent chlorate treated columns, and blue bars represent nitrate treated columns. **(A)** Inhibitory effects of each treatment. **(B)** Enrichment effect due to each treatment.

To investigate the specific inhibitory and stimulatory impact of the treatments relative to the inoculum, we compared the relative abundance of the top 10% of the OTUs contributing to the differences between the treated columns, the untreated columns, and the original inoculum. A number of different phyla were enriched or inhibited by the individual treatments compared to the inoculum (Figure S3 and Figures 4A,B). In the absence of any treatment a few families from the phylum Firmicutes and Deltaproteobacteria, specifically the canonical sulfate reducing families *Desulfovibrionaceae* and *Desulfobacteraceae* (Figure S3) were stimulated to a small extent relative to the original inoculum. Similarly, several Proteobacterial OTUs decreased with the largest change observed in the *Oceanospirillaceae* and *Vibrionaceae* families (Figure S3). In the case of *Oceanospirillaceae* this is not surprising as these families are composed primarily of aerobic respirers (Garrity et al., 2005) while *Vibrionaceae* tend to be symbionts of marine animals although there are many free-living species capable of fermentation. Both perchlorate and chlorate had a general inhibitory impact across a broad range of phyla with a minimal stimulatory impact on any individual phylum compared to the inoculum suggesting a broad suppression of microbial activity. Of interest were the differences observed between perchlorate and chlorate treatments, which may be influenced by the disparity in their relative chemical reactivity (Figure S1) (Urbansky, 1998, 2002; Urbansky and Brown, 2003). Various Proteobacteria were of lower relative abundance in perchlorate treatments with the *Vibrionaceae* being particularly affected (Figure 4A). Other less abundant phyla in perchlorate treatments included Acidobacteria, Verrucomicrobia, Gemmatimonadetes, OP8, Planctomycetes, and WS3. Chlorate affected many, but not all, of the same phyla (Figure 4A).

**Figure 5:**
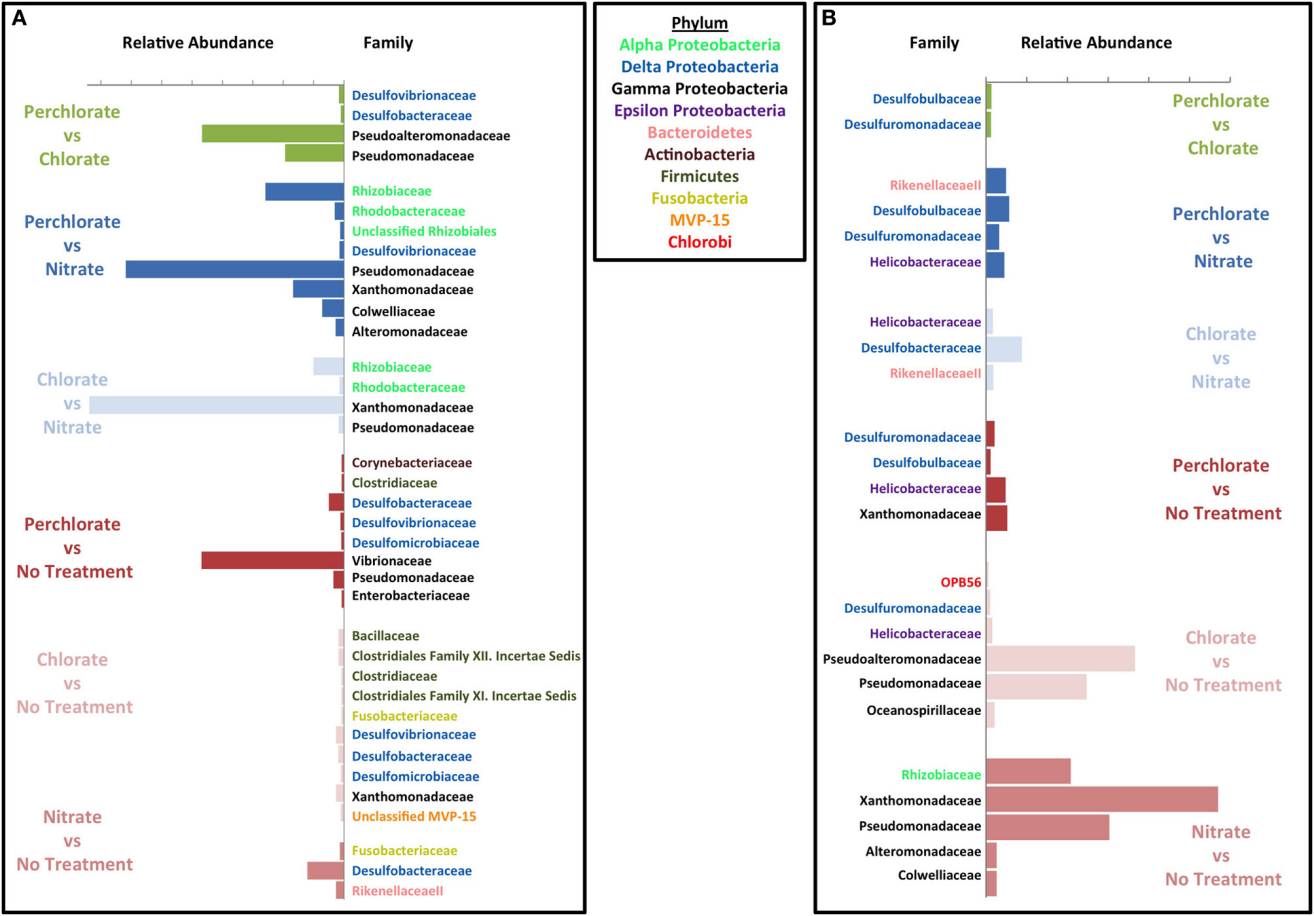
**Effect of each treatment on microbial community compared to the other treatments**. Similarity percentage (SIMPER) was used to determine the OTUs that contribute to the top 10% of difference between each set of treatment samples and the initial innoculum samples. Average abundance for the OTUs belonging to the same family were added together. Abundance on the x axis is the difference between average abundance for samples from the corresponding treatment and the initial innoculum samples. Each plot is labeled by family on the y axis and the family names are color coded by phylum. Green bars represent comparison between perchlorate and chlorate treated columns, blue bars represent comparisons against nitrate treated columns (with the perchlorate vs. nitrate in dark blue and chlorate vs. nitrate in light blue), and red bars represent comparisons against untreated columns (Perchlorate vs. no treatment in dark red, chlorate vs. no treatment in light red and nitrate vs. no treatment in medium red). **(A)** Inhibitory effects. **(B)** Enrichment effects.

In contrast to its inhibitory effects, perchlorate enriched for various members of the Firmicutes, Fusobacteria, Proteobacteria (Delta, Gamma, and Epsilon), Verrucomicrobia, Euryarcheota, and Tenericutes (Figure 4B). Chlorate also enriched for some members of the Proteobacteria (Delta, Gamma, and Epsilon), Firmicutes, Bacteroidetes, and Tenericutes. Curiously, of these various phyla only the Proteobacteria (Alpha, Beta, Gamma, and Epsilon), and Firmicutes have previously described (per)chlorate reducing members with the Betaproteobacteria containing the environmentally dominant perchlorate reducing genera (*Dechloromonas* and *Azospira* species) and Gammaproteobacteria containing the majority of the known chlorate reducing species (*Pseudomonas*) (Coates and Achenbach, 2004). In support of this observation, chlorate treatment specifically stimulated members of the *Pseudomonadaceae* (Figures 4B, 5B). Interestingly, perchlorate did not enrich for Betaproteobacteria. However, this may be a function of the unsuitability of marine conditions to either the *Dechloromonas* or *Azospira* genera, neither of which contain halotolerant isolates (Coates and Achenbach, 2004).

In contrast to both perchlorate and chlorate, while nitrate treatment did have some inhibitory effect on members of the microbial community relative to the inoculum, there was also a strong enrichment of specific phyla (Figures 4A,B). This result supports the conclusions drawn based on the evenness and diversity indices above. Nitrate inhibited members of the Acidobacteria, Gammaproteobacteria, Bacteroidetes, Chloroflexi, Gemmatimonadetes, Planctomycetes, and Verrucomicrobia phyla, while it enriched for Proteobacteria (Alpha, Gamma, and Delta), Firmicutes, and Tenericutes; all of which contain members known to perform nitrate reduction. Nitrate treatment specifically enriched for members of the families *Xanthamonadaceae* and *Pseudomonadaceae* within the Gammaproteobacteria. These families contain ubiquitous members known as canonical nitrate reducing organisms (*Xanthamonas* and *Pseudomonas* species).

Comparisons were also made between the different treatments (Figures 5A,B). When compared to the no treatment control, all treatments (nitrate, perchlorate, and chlorate) inhibited known sulfate reducing genera supporting the observed geochemical results. Interestingly, both perchlorate and chlorate showed an inhibitory impact on several known sulfate reducing families (*Desulfovibrionaceae, Desulfobacteraceae*, and *Desulfomicrobiaceae*), while nitrate only showed inhibition of the *Desulfobacteraceae* relative to the control reactors (Figure 5A). This result supports the isotopic and geochemical evidence indicating that sulfate reduction occurred in the nitrate-amended columns but was masked by the activity of sulfur oxidizing nitrate reducers.

Both chlorate and nitrate had a stimulatory effect on specific genera (chlorate: *Pseudomonadaceae* and *Pseudoalteromonadaceae*; nitrate: *Pseudomonadaceae, Xanthamonadaceae*, and *Rhizobiaceae*) relative to the no treatment control (Figure 5A) suggesting that members of these families were responsible for chlorate and nitrate respiration, respectively. In contrast, perchlorate did not result in any obvious stimulatory effect in comparison to the no treatment control and only four families were stimulated to any degree (*Desulfuromonadaceae, Desulfobulbaceae, Helicobacteraceae*, and *Xanthamonadaceae*). This result further suggests that community adaptation to perchlorate was slow relative to its inhibitory effect as a whole and diverse growth was not associated with its presence. The result also suggests that members of one or more of the *Desulfuromonadaceae, Desulfobulbaceae*, and *Xanthamonadaceae* families have the previously unrecognized ability to respire perchlorate. *Helicobacteraceae* includes the known perchlorate reducer *Wolinella succinogenes*. Relative to the chlorate treatment, perchlorate had the greatest inhibitory impact on members of the Deltaproteobacteria and Gammaproteobacteria (Figure 5A). The inhibited Deltaproteobacteria represent the canonical sulfate reducing families *Desulfovibrionaceae* and *Desulfobacteraceae* suggesting that perchlorate may be more effective than chlorate as an inhibitor of the SRM in the community. A similar comparison with nitrate also indicated that perchlorate showed a greater inhibition of members of the *Desulfovibrionaceae*, a result that is supported by geochemical evidence (Figure 1C). The inhibition of Gammaproteobacteria by perchlorate relative to chlorate may be explained by the selective enrichment in this phylum by chlorate treatment relative to the perchlorate treatment and the no treatment control (Figure 5B).

## Discussion

Our study clearly demonstrates that both perchlorate and chlorate are effective inhibitors of microbial sulfate reduction in a marine system. This conclusion is drawn from geochemical, isotopic, and 16S rRNA community data analysis. In the absence of any treatment, rapid and extensive sulfate reduction was observed in flow through column systems. However, the presence of either perchlorate or chlorate effectively constrained sulfide production.

In the case of nitrate treatment, although sulfide production was initially attenuated, nitrate was completely consumed in the columns and measurable sulfide reappeared 23 days into the study suggesting that microbial community succession was occurring as previously observed (Voordouw et al., 1996; Myhr et al., 2002; Bødtker et al., 2008, 2009; Hubert, 2010; Callbeck et al., 2011). Both the stable isotope and community analyses support this result and suggest that the true extent of SRM activity was masked through the activity of nitrate-dependent sulfur oxidizing (NDSO) microorganisms as previously suggested (Telang et al., 1997; Greene et al., 2003; Hubert et al., 2009; Hubert, 2010). Complete conversion of sulfide to sulfate by NDSO activity allows for sulfur cycling to occur between sulfide and sulfate species which can maintain active SRM populations in the presence of nitrate, especially if the organisms are resistant to nitrite that may be produced as a transient intermediate of nitrate respiration (Greene et al., 2003; He et al., 2010). In electron donor rich systems such as oil reservoirs, which are often replete with biologically labile organic acids (Vance and Thrasher, 2005) and hydrocarbons that many SRM are capable of utilizing (Aeckersberg et al., 1991; Beller et al., 1992, 1996; Edwards et al., 1992; Widdel et al., 1992; Lovley et al., 1995; Coates et al., 1996a,b, 1997; Bedessem et al., 1997; Caldwell et al., 1998; Galushko et al., 1999; Anderson and Lovley, 2000; Annweiler et al., 2000; Abu Laban et al., 2009), such sulfur redox cycling combined with heterotrophic nitrate reduction can lead to a more rapid depletion of the nitrate with the resultant onset of uninhibited sulfate reduction deeper in a reservoir (Callbeck et al., 2011). Furthermore, as some SRM are alternatively capable of utilizing nitrate as a suitable electron acceptor (Keith and Herbert, 1983; Dalsgaard and Bak, 1994; Moura et al., 1997, 2007; López-Cortés et al., 2006; Marietou et al., 2009), nitrate addition may in fact enhance population size rather than limiting it resulting in a robust SRM community that can generate large amounts of sulfide if nitrate injection is interrupted, such as during reservoir shut-in periods. No SRM have been isolated to date that can grow by respiratory perchlorate reduction, though some evidence exists that at high temperatures a cryptic mixed biotic-abiotic perchlorate reduction process can be catalyzed by thermophilic sulfate reducers (Liebensteiner et al., 2013).

In contrast to the nitrate and control columns, no sulfide was detected in effluent from either the perchlorate or chlorate columns unless the treatment concentration was dropped to 5 mM. Even in this instance, sulfide production was immediately eradicated on reestablishment of the treatment concentration suggesting a concentration threshold may exist. The nature of this threshold concentration is likely to be a function of the inhibitory effect of (per)chlorate on microbial sub-populations and the preferential thermodynamics of (per)chlorate respiration.

The sulfur isotope data showed greater evidence of dynamic sulfur cycling than was evident from the fluid chemistry alone. The combination of low δ^34^S sulfate values and elevated sulfide in the nitrate treatment is consistent with active sulfur redox cycling in the nitrate columns in which the δ^34^S isotopic signature of sulfate reduction is (partially) masked by the microbial oxidation of sulfide to sulfate by NDSO microorganisms as previously observed (Hubert et al., 2009). This is because, in comparison with microbial sulfate reduction, microbial sulfide oxidation often produces little sulfur isotope fractionation (Toran and Harris, 1989; Hubert et al., 2009; Brabec et al., 2012). As such, the sulfate produced retains low δ^34^S values similar to the sulfide, thus the net sulfate in the effluent is a combination of the heavy residual sulfate from sulfate reduction (high δ^34^S values) and the light biogenic sulfate from sulfide oxidation (low δ^34^S values). Ultimately, in a nitrate treated column, effluent sulfate δ^34^S will depend on the relative importance of the biocompetitive exclusion mechanism and the sulfide re-oxidation pathway, as seen previously by Hubert et al. (2009). In comparison, although DPRB oxidize sulfide innately, the end product of this metabolism is generally insoluble elemental sulfur (Gregoire et al., 2014) which would remove sulfide from the system without affecting the δ^34^S values of the residual sulfate in the effluent. The sulfur isotope data also (Figure 2) suggested that even when sulfide was not detected in the column effluent, some residual sulfate reduction was occurring in the treated columns, demonstrating that the δ^34^S of effluent sulfate in oil reservoir production waters could potentially provide an effective early indication of souring before breakthrough of the H_2_S front in reservoirs where the rock matrix has significant H_2_S scavenging potential.

Our microbial community data illustrated that each treatment had a distinct impact on the overall microbial community. Nitrate treatment caused the most dramatic shift in community composition as assessed by nMDS analysis and hierarchical clustering. This is not surprising because complete nitrate reduction to ammonia or dinitrogen involves multiple genes (*nir, nos, nrf*) that are present in a broad diversity of bacterial phylotypes in mixed microbial ecosystems (Moreno-Vivián et al., 1999). Thus, nitrate addition is expected to stimulate a mixture of bacterial families from across the phylogenetic tree.

Deltaproteobacteria are an important component of the microbial communities in this study. The Deltaproteobacteria include both SRMs and non-SRMs. The SRMs fall into various families including *Desulfovibrionaceae, Desulfobulbaceae, Desulfomicrobiaceae*, and *Desulfobacteriaceae*. Elemental sulfur (S^0^) reducing members of this class include *Desulfurellaceae, Pelobacteraceae*, and *Desulfuromonadaceae* (Castro et al., 2000; Friedrich et al., 2001). *Desulfobulbaceae* species have also been shown to be capable of growing by sulfur oxidation (Pfeffer et al., 2012). Although Callbeck et al. (2011) showed *Desulfobulbus* (family *Desulfobulbaceae*) to be primarily SRMs, in our reactors they may act as elemental sulfur reducers or sulfide oxidizers because members of this family are primarily enriched in treated columns where no net sulfide production is observed. Obligately S^0^ reducing families (*Desulfuromonadaceae)* are also enriched in (per)chlorate treatments vs. both nitrate treatment and the no treatment controls. Based on known metabolic capabilities of members of the *Desulfobulbaceae* (sulfate reduction, S^o^ disproportionation, and autotrophic H_2_S oxidation) and the *Desulfuromonadaceae*, the enrichment of these organisms may involve the redox cycling of sulfur between H_2_S and elemental sulfur which supports earlier studies demonstrating that all DPRB innately oxidize H_2_S to elemental sulfur (Gregoire et al., 2014). In support of a central role of elemental sulfur in these reactors, (per)chlorate treatments also enriched for *Helicobacteraceae* which includes members of the genera *Flexispira, Sulfurimonas, Sulfurovum*, and *Sulfuricurvum*, all of which are known to oxidize sulfur and all of which were enriched by perchlorate (Inagaki et al., 2003, 2004; Kodama and Watanabe, 2004; Takai et al., 2006). Isolated perchlorate reducing bacteria all belong to the Proteobacteria (Alpha, Beta, Gamma, and Epsilon) and Firmicutes. All isolated chlorate-reducing bacteria are Proteobacteria (Beta, and Gamma). However, all perchlorate reducing organisms can also reduce chlorate (Coates and Achenbach, 2004) and evidence indicates the ability to respire both perchlorate and chlorate has been spread by horizontal gene transfer (Coates and Achenbach, 2004; Melnyk et al., 2011, 2014; Clark et al., 2013) suggesting that the diversity of organisms capable of these metabolisms may be far more extensive than current isolates suggest. This may explain why (per)chlorate treatment enriches for a variety of Firmicutes, Fusobacteria, Gammaproteobacteria, Bacteroidetes, and Epsilonproteobacteria.

Despite the fact that no sulfide was measured in the effluent from the (per)chlorate columns both the isotopic measurements and community analysis support the fact that (per)chlorate indeed stimulates sulfur cycling in the column system. (Per)chlorate treatment enriches for organisms known to be capable of sulfide oxidation, S^0^ oxidation, S^0^ disproportion, and S^0^ reduction; and results in a decrease in the abundance of canonical SRM. This supports a model in which perchlorate both directly inhibits sulfate reduction and stimulates sulfur cycling. Nitrate is not only less effective at preventing souring in this system, but it also doesn't appear to stimulate the same sort of sulfur cycling that is seen in the (per)chlorate columns.

Taken together our findings indicate that (per)chlorate and nitrate are mechanistically distinct inhibitors of sulfide production in complex natural ecosystems. The apparently greater inhibitory potency of (per)chlorate relative to nitrate and potentially favorable shifts in geochemistry and microbial communities suggest that (per)chlorate may be a promising alternative to nitrate for controlling bio-souring in industrial ecosystems. Currently, the potential for perchlorate respiration in an oil reservoir is unknown. Future studies are needed to investigate the application of perchlorate with sulfidogenic samples from industrial ecosystems including oil reservoirs using crude oil or produced water organic components as the electron donor(s).

### Conflict of interest statement

The corresponding author has an IP submission based on souring control. The Associate Editor M. Youngblut declares that despite being affiliated to the same institution as the author(s) J. D. Coates, the review process was handled objectively and no conflict of interest exists. The authors declare that the research was conducted in the absence of any commercial or financial relationships that could be construed as a potential conflict of interest.
