# Supplementary material for: Inhibition of microbial sulfate reduction in a flow-through column system by (per)chlorate treatment
Source: Front Microbiol. 2014 Jun 26;5:315. doi: 10.3389/fmicb.2014.00315 (PMC4092371; doi:10.3389/fmicb.2014.00315)
Supplement: Supplementary file 1 [file Presentation1.PDF]

Fig S1

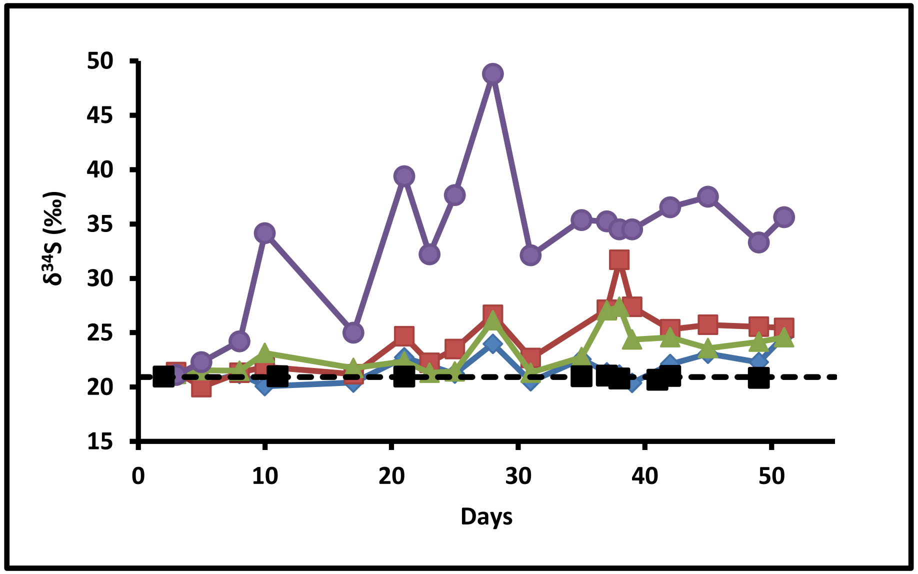

Fig S2

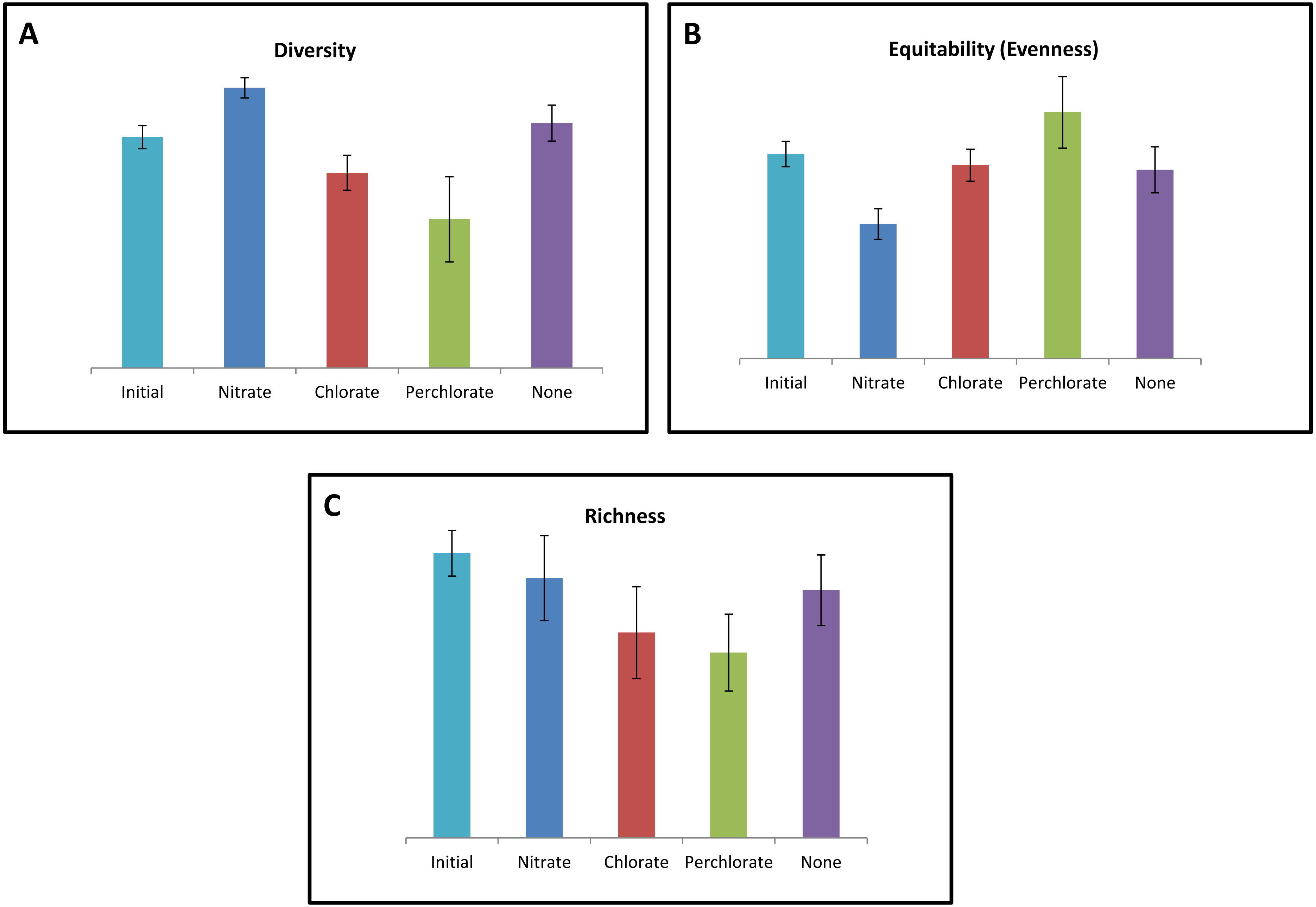
A
Diversity
Initial
Nitrate
Chlorate
Perchlorate
None
B
Equitability (Evenness)
Initial
Nitrate
Chlorate
Perchlorate
None
C
Richness
Initial
Nitrate
Chlorate
Perchlorate
None

Fig S3

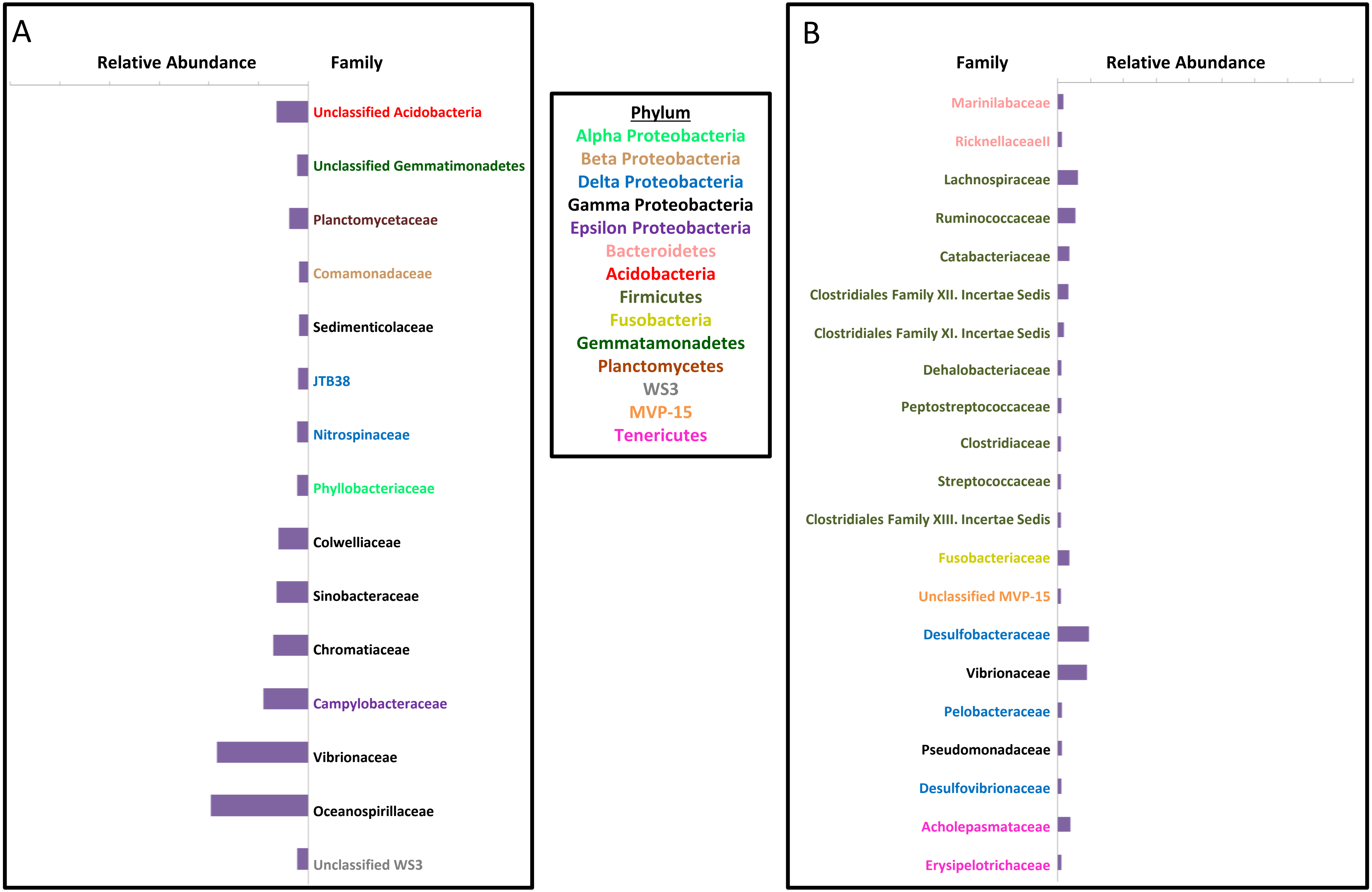
A
Relative Abundance
Family
Unclassified Acidobacteria
Unclassified Gemmatimonadetes
Planctomycetaceae
Comamonadaceae
Sedimenticolaceae
JTB38
Nitrospinaceae
Phyllobacteriaceae
Colwelliaceae
Sinobacteraceae
Chromatiaceae
Campylobacteraceae
Vibrionaceae
Oceanospirillaceae
Unclassified WS3
Phylum
Alpha Proteobacteria
Beta Proteobacteria
Delta Proteobacteria
Gamma Proteobacteria
Epsilon Proteobacteria
Bacteroidetes
Acidobacteria
Firmicutes
Fusobacteria
Gemmatamonadetes
Planctomycetes
WS3
MVP-15
Tenericutes
B
Family
Relative Abundance
Marinilabaceae
RicknellaceaeII
Lachnospiraceae
Ruminococcaceae
Catabacteriaceae
Clostridiales Family XII. Incertae Sedis
Clostridiales Family XI. Incertae Sedis
Dehalobacteriaceae
Peptostreptococcaceae
Clostridiaceae
Streptococcaceae
Clostridiales Family XIII. Incertae Sedis
Fusobacteriaceae
Unclassified MVP-15
Desulfobacteraceae
Vibrionaceae
Pelobacteraceae
Pseudomonadaceae
Desulfovibrionaceae
Acholepasmataceae
Erysipelotrichaceae

Supplementary Figure Legends

Fig S1: Abiotic reactivity of Fe(II) incubated for 150 hours with 10 mM nitrate (blue diamonds), chlorate (red squares), or perchlorate (green triangles). Anoxic bicarbonate buffered basal medium (BBM), pH 6.8 at 37 °C was amended with 10 mM sodium nitrate, 10 mM sodium perchlorate or 10 mM sodium chlorate. Addition of 10 millimolar Fe(II) to the growth medium immediately formed a vivianite white precipitate leaving 1-2 mM soluble Fe(II). Soluble and insoluble Fe(II) were measured separately to characterize the oxidation of each Fe(II) form in cultures and to avoid analytical artifacts due to rapid reactions which occur between NO_2^- and Fe(II) in acidic solution and the regeneration of NO_2^- through reactions between NO and O_2 under oxic conditions. At each time point, 0.5 mL of culture media was anaerobically removed and centrifuged to separate insoluble Fe(II) from soluble Fe(II) and NO_2^- in solution. Soluble Fe(II) was directly measured with the ferrozine assay and insoluble Fe(II) was resuspended in 0.5 M HCl for 24 hrs and quantified using the ferrozine assay.

In the column experiment, intrinsic Fe(II) would similarly act as a catalyst in the presence of H_2S and chlorate. The putative reaction scheme is outlined below.

$6FeOOH + 3HS^- + 15H^+ \rightarrow 6Fe(II) + 3S^o + 12H_2O$ ………………………… 1

This reaction will result in the reduction of the ferric iron to ferrous [Fe(II)] iron. However, Fe(II) will chemically react with chlorate (ClO_3^-) becoming reoxidized back to Fe(III) according to reaction 2:

$ClO_3^- + 6Fe^{2+} + 9H_2O \rightarrow 6FeOOH + Cl^- + 12H^+$ …………………………….. 2

Thus, the net of these two reactions is the abiotic oxidation of H_2S coupled to the reduction and consumption of chlorate as shown for reaction 3 below.

$ClO_3^- + 3HS^- + 3H^+ \rightarrow 3S^o + Cl^- + 3H_2O$ …………………………………….. 3

As can be seen from Fig. S1, the kinetics of reaction are of the same order of magnitude as that of the biological oxidation of H_2S coupled to chlorate.

Fig S2: A. Relative diversity for each treatment calculated from the PhyloChip abundance data with Y axis labels removed to emphasize relative values. B. Relative evenness for each treatment calculated from the PhyloChip abundance data. C. Sample richness calculated from PhyloChip presence / absence data.

Fig S3: Effect of no treatment on microbial community compared to the initial innoculum. Similarity percentage (SIMPER) was used to determine the OTUs that contribute to the top 10% of difference between each set of treatment samples and the initial inoculum samples. Average abundance for the OTUs belonging to the same family were added together. Abundance on the x axis is the difference between average abundance for samples from the corresponding treatment and the initial inoculum samples. Each plot is labeled by family on the y axis and the family names are color coded by phylum. A. Inhibitory effects of each treatment. B. Enrichment effect due to each treatment
